# Smart Optimization Method for Safety Signs in Innovative Manufacturing Environments Integrating Industrial Field IoT Sensors and Knowledge Graphs

**DOI:** 10.3390/s26123965

**Published:** 2026-06-22

**Authors:** Yixiang Li, Jianxin Chen, Jing Yang

**Affiliations:** 1 School of Art and Design, Wuhan University of Technology, Wuhan 430070, China; l2686128631@126.com; 2 School of Innovation & Design, Wuhan Textile University, Wuhan 430073, China

**Keywords:** knowledge graph, innovative manufacturing environment, intelligent optimization of safety labels, IoT sensor, industrial field

## Abstract

**Highlights:**

**What are the main findings?**

- A dual-channel fusion reasoning framework integrates DGAT and a hierarchical temporal knowledge graph for industrial safety.
- Dynamic adjacency matrix with time-varying spatial coordinates adapts sensor topology to layout rearrangement in real time.

**What is the implication of the main finding?**

- Reinforcement learning with composite reward function achieves closed-loop control from perception to adaptive signage expression.
- Real-world validation on an aerospace workshop confirms the effectiveness of the proposed method for risk identification and response latency.

**Abstract:**

Safety signs in innovative manufacturing environments fail to match dynamic risks due to the separation of perception, semantics, and decision-making. Existing methods lack closed-loop integration of IoT sensor streams, knowledge graph reasoning, and adaptive signage control. This paper proposes a framework that fuses dynamic graph attention networks with hierarchical temporal knowledge graphs and reinforcement learning optimization. The framework extracts spatiotemporal dependencies from multi-source sensors, traces risk propagation paths on an industrial knowledge graph, and generates adaptive signage actions. Experimental results demonstrate that the proposed method achieves 96.7% risk identification accuracy, a 91.3% risk propagation F1 score, a 94.2 semantic matching score, and 43.65 milliseconds response latency. Real-world validation on an aerospace workshop confirms the method’s effectiveness. This work provides a closed-loop solution from physical perception to adaptive semantic expression for intelligent manufacturing safety.

## 1. Introduction

The current industrial safety identification system generally adopts a static preset or single sensor threshold-triggered working mode, lacking a closed-loop mechanism for organically integrating real-time perception data of the Internet of Things with deep semantic logic of industrial knowledge graphs and transforming them into adaptive identification expressions. In innovative manufacturing environments such as flexible production lines, temporary workstations, and dynamic task collaboration, the frequent adjustment of production line layout, fluctuations in workstation task variability, and randomization of personnel flow paths are intertwined with dynamic factors, resulting in the inability to accurately match the warning content, presentation intensity, and spatial projection boundaries of safety signs with the real-time evolution of on-site risks [1,2]. Existing systems often only passively alert after accidents occur, making it difficult to actively adjust identification strategies during the risk incubation stage to guide personnel behavior avoidance [3]. The fundamental reason for this deficiency is that the physical signals extracted by the perception layer have not been dynamically associated with the rule knowledge contained in the semantic layer, and the decision layer lacks explicit modeling of environmental dynamic parameters, resulting in a disconnect between the control logic of the identification terminal and the actual disturbance state of the manufacturing environment [4]. Designing an intelligent identification optimization method has become a key technical requirement for improving safety protection in innovative manufacturing environments. This method must deeply integrate sensor spatiotemporal characteristics with knowledge graph semantic reasoning and must possess environmental adaptation capability. Industrial digital twins and cyber–physical system safety frameworks provide a holistic representation of manufacturing assets and their dynamic behaviors under evolving operational conditions. A digital twin of an innovative manufacturing environment maintains a synchronized virtual replica of physical layouts, sensor deployments, workstation tasks, and personnel flows, enabling real-time risk simulation and predictive safety assessment. Cyber–physical system safety frameworks extend beyond traditional monitoring by embedding closed-loop control logic that directly couples physical sensing with semantic decision-making. Edge AI manufacturing systems push computational intelligence from cloud servers to on-site controllers and gateway devices, reducing the latency of data processing and inference while preserving the contextual awareness required for rapid risk response. Industry 4.0 semantic architectures organize manufacturing knowledge through ontologies and knowledge graphs that standardize device descriptions, process definitions, and hazard taxonomies across heterogeneous production modules. The proposed method aligns with these paradigms by deploying dynamic graph attention network inference on edge nodes, using a knowledge graph that embodies semantic architecture principles, and integrating sensor streams with a digital twin simulation environment built on ROS and Gazebo. Modern graph reasoning systems including temporal graph networks and graph Transformers have advanced the capability to model evolving relational structures in industrial settings. Temporal graph networks maintain memory modules for each node to capture long-range historical interactions but require multiple observation steps to adapt to abrupt topological changes such as layout rearrangement. Graph Transformers apply self-attention mechanisms over graph nodes but scale quadratically with the number of nodes and lack explicit modeling of spatiotemporal decay based on physical distances. The proposed dual-channel framework addresses these limitations through a dynamic adjacency matrix that recalibrates sensor correlations instantaneously using time-varying spatial coordinates and time-frequency feature similarity. The hierarchical attention temporal knowledge graph inference module further incorporates static rule embeddings from the semantic architecture and bidirectional gated recurrent unit temporal encodings, enabling rapid risk propagation path tracing without the convergence lag of memory-based methods. This design positions the proposed method within the broader context of edge AI manufacturing systems and Industry 4.0 semantic architectures while offering distinct advantages over existing modern graph reasoning approaches in dynamic manufacturing environments.

The specific problem objects addressed in this article include three types of coupled dynamic elements. The first type is a multi-source heterogeneous IoT sensor node group. This group covers vibration accelerometers, infrared temperature sensors, industrial cameras, and gas concentration detectors. These sensors undergo spatial changes when the production line layout is rearranged. The equipment’s abnormal states and personnel’s dangerous behavior patterns contained in their timing signals require real-time extraction [5,6]. The second is the dynamic parameter set of the manufacturing environment, including layout change identification, workstation task changes, and personnel flow. These parameters are independent of sensor observations but affect the spatial propagation path of risks and the hazard perception threshold of personnel [7,8]. The third is the security identification terminal cluster, consisting of laser projection units, electronic screen arrays, and wearable tactile devices. Its controllable actions include projection area boundary coordinates, display content index, and vibration intensity level [9,10]. The importance of the above three types of objects lies in the fact that only by simultaneously capturing the spatiotemporal dependencies of physical signals, the statistical characteristics of environmental disturbances, and the physical constraints that identify actuators, can a complete closed loop be constructed from “what risks are perceived” to “how to express warnings”. Neglecting any of these links can result in semantic mismatch or response failure of the identification strategy under dynamic operating conditions.

A series of algorithms and models have been proposed from different perspectives to address the above issues. The traditional static identification threshold method is based on a fixed sensor threshold triggering preset content, which is simple to implement but cannot handle multi-factor coupling risks. Its accuracy deteriorates severely after layout changes [11]. The sequence model based on CNN-LSTM (Convolutional Neural Networks–Long Short-Term Memory) can extract deep features of temporal signals, predict risk types, and report their security risks. However, it relies on data-driven approaches and lacks domain knowledge constraints, resulting in insufficient inference ability for rare or complex risk events [12,13]. The static graph convolutional network knowledge inference method propagates rules on a pre-constructed security knowledge graph, which can trace the causal chain of accidents, but the sensor embeddings are fixed as static attributes [14,15]. Some studies have attempted to introduce multimodal and deep learning optimization for risk identification and warning, achieving fast and real-time response [16]. However, existing methods generally suffer from the problem of the three-stage separation of perception, semantics, and decision-making: either sensor features are not injected into the knowledge graph in real time, or the graph inference results do not drive the adaptive adjustment of identification terminals, or the decision model lacks explicit modeling of environmental dynamics, making it difficult to achieve accurate, real-time, and robust intelligent identification optimization in the complex disturbances of innovative manufacturing environments. Existing architectures fail because their perception, semantic, and decision stages operate on fixed or slowly updated representations that do not adapt to the temporal and spatial dynamics inherent in innovative manufacturing environments. The perception stage in static threshold methods and CNN-LSTM models extracts features from sensor signals without integrating the evolving physical topology caused by layout rearrangement, leading to a mismatch between detected anomalies and their spatial origins. The semantic stage in static graph convolutional networks propagates rules over a knowledge graph whose node embeddings remain frozen as initial static attributes, preventing the graph from reflecting runtime changes in workstation configurations and material flow paths. The decision stage in all baseline architectures treats environmental parameters such as layout change identifiers, task variation rates, and personnel entropy as unmodeled external noise rather than explicit state variables, so the identification terminal control logic cannot anticipate risk propagation shifts induced by these disturbances. This sequential decoupling with static representations and omitted environmental variables produces a cascading failure mode where each stage’s errors amplify the next stage’s misalignment with the true dynamic risk landscape.

The research objective of this article is to construct a fully closed-loop security identification system from physical perception to adaptive semantic expression, achieving millisecond level collaborative optimization of risk perception and identification expression in dynamic environments. Therefore, a dual channel fusion inference framework based on spatiotemporal dynamic graph neural network and hierarchical temporal knowledge graph is proposed. Firstly, a joint architecture of a dynamic graph attention network and short-time Fourier transform is designed in the perception layer to convert multi-source sensor temporal signals into dynamic adjacency matrices. Deep spatiotemporal dependencies are extracted through multi-channel graph attention to generate high-dimensional sensing semantic vectors. Secondly, a hierarchical attention temporal knowledge graph inference model is introduced in the cognitive layer to inject sensing semantic vectors into the industrial safety knowledge graph through entity binding. A joint embedding mechanism of a graph convolutional network and bidirectional gated recurrent unit is used to align static rules and dynamic features. The risk propagation path is tracked hop by hop on the graph structure and the risk semantic vector is output. Finally, a hierarchical semantic risk-mapping and reinforcement learning-driven dynamic identification optimization algorithm is constructed at the decision-making end. The risk’s semantic vector and the augmented state of the environment’s dynamic encoding are used as inputs, and the controllable parameters of the identification terminal are used as the action space. A composite reward function that integrates safety accident punishment and behavior compliance change rate feedback is designed, and the optimal identification strategy network is trained iteratively through Q-learning. The experimental results on the innovative manufacturing environment simulation platform based on the ROS (Robot Operating System) and Gazebo show that the risk identification accuracy of this method reaches 96.7%, and the dynamic identification response delay is as low as 43.65 milliseconds. The F1 value of risk propagation path inference reached 91.3%, and the matching score between identification semantics and risk scenarios was 94.2 points, demonstrating performance advantages compared to existing methods in dynamic working conditions.

## 2. A Closed-Loop Optimization Method for Security Identification That Integrates Perception and Semantic Reasoning

### 2.1. Overall System Framework and Definition of Dynamic Environment Data Flow

The closed-loop optimization system constructed in this article consists of three cascaded layers. These layers are the perception layer, the cognitive layer, and the expression layer. Its data flow definition revolves around explicit modeling of environmental dynamic parameters. At time t, the system input is the multi-source sensor observation matrix $X_{t}$, where N is the total number of sensor nodes and L is the number of sampling points within the sliding window [17,18]. Define the dynamic parameter vector $e_{t}\;=\;[\delta_{t},\rho_{t},\eta_{t}{]}^{⊤}$. The term $\delta_{t}$ is the layout change identifier, and when taken as 1, it indicates that the current layout rearrangement window period is in progress; $\rho_{t}$ is the variation rate of workstation tasks, defined as the ratio of the number of workstations with task type changes occurring within the sliding window to the total number of workstations. The term $\eta_{t}$ is the entropy value of personnel mobility, calculated from the normalized information entropy of personnel trajectory direction distribution. This vector is independent of sensor observations and is injected into each layer module as a global context. The innovative manufacturing environment studied in this article refers to a type of production space with high dynamic reconfigurability and task uncertainty. Unlike traditional manufacturing environments with fixed, steady-state processes, the core features of this environment include rearrangement of physical layout, high variability of workstation tasks, and personnel flow. These dynamic elements are coupled with each other, making it difficult for static preset models to capture the spatial propagation boundaries and temporal evolution laws of security risks.

The perception layer performs spatiotemporal feature extraction of sensor data. For the i-th sensor node, take the temporal signal $x_{i}(t\;-\;L\;+\;1:t)$ within the window, and obtain the time-frequency feature matrix $H_{i}^{\left(t\right)}$ through short-time Fourier transform, where t is the current time step [19,20]. The three-dimensional physical coordinates of node $v_{i}$ at time t are defined as $p_{i}(t)$. These coordinates undergo a step update when a layout rearrangement event occurs to capture sensor spatial topology evolution caused by layout changes in innovative manufacturing environments. The element $a_{\mathrm{ij}}^{\left(t\right)}$ in the dynamic adjacency matrix $A_{t}$ is constructed as follows:

(1) $$a_{\mathrm{ij}}^{\left(t\right)}=\sigma \left(\frac{h_{i}^{\left(t\right)}\cdot h_{j}^{\left(t\right)}}{\|h_{i}^{\left(t\right)}{\|}_{2}\|h_{j}^{\left(t\right)}{\|}_{2}}\cdot \exp\left(-\lambda \|p_{i}(t)-p_{j}(t){\|}_{2}\right)\right)$$

In Formula (1), $h_{i}^{\left(t\right)}=\mathrm{vec}(H_{i}^{\left(t\right)})$ is the vectorized expansion of the time-frequency feature matrix, where σ(·) is the Sigmoid activation function and λ is the spatial distance attenuation coefficient, which controls the degree of penalty of physical proximity on correlation strength. This formula automatically recalibrates the functional coupling relationship between sensors as the layout changes. Input $A_{t}$ and the node feature matrix into a stacked multi-channel dynamic graph attention network. After K-layer graph attention aggregation, output the sensing semantic vector $z_{t}$, whose dimension $d_{z}$ is determined by the number of output channels in the last layer [21,22].

The cognitive layer receives $z_{t}$ and $e_{t}$ to drive hierarchical temporal knowledge graph inference. The knowledge graph G = (ℰ, ℛ, T) includes static entity sets, relationship sets, and temporal fact sets, and expands dynamic entity subclasses such as temporary workstations and temporary collaborative tasks [23,24]. The sensing semantic vector injects corresponding physical node attributes through entity binding rules: for the bound node $e_{k}$, its attribute vector is updated to $v_{k}^{\left(t\right)}=\;MLP([v_{k}^{\left(0\right)}⊕z_{t}^{\left(k\right)}])$, where $v_{k}^{\left(0\right)}$ is the initial static embedding, $z_{t}^{\left(k\right)}$ is the block corresponding to $e_{k}$ in $z_{t}$, and ⊕ is the vector concatenation. On this basis, a joint embedding mechanism of a graph convolutional network and bidirectional gated recurrent unit is introduced [25,26], and the update rule of node $e_{i}$ in the l-th layer GCN (Graph Convolutional Network) is defined:

(2) $$u_{i}^{\left(l\right)}=\mathrm{ReLU}\left(\sum_{r}\sum_{j}\frac{1}{c_{i,r}}W_{r}^{\left(l\right)}v_{j}^{(l-1)}+W_{0}^{\left(l\right)}v_{i}^{(l-1)}\right)$$

In Formula (2), $c_{i,r}$ is normalization constant, r represents the type of relationship in the knowledge graph, $W_{r}^{\left(l\right)}$ and $W_{0}^{\left(l\right)}$ are learnable parameter matrices, and i and j are index variables. At the same time, the time-series snapshot sequence is input into Bi GRU (Bidirectional Gated Recurrent Unit) to capture the evolutionary trend of the risk state. After the fusion of static embedding and dynamic embedding through attention gating, a risk propagation path search is performed on the graph structure, and the output risk semantic vector $r_{t}\;=\;[\gamma_{t},c_{t},b_{t}{]}^{⊤}$, where $\gamma_{t}$ is the risk quantification level, $c_{t}$ is the risk type encoding, and $b_{t}$ is the boundary parameterized representation of the affected spatial region.

The expression layer concatenates $r_{t}$ and $e_{t}$ into an augmented state vector $s_{t}\;=\;[r_{t}⊕e_{t}]$, which serves as the input for the Optimal Signage Policy Network (OSPN) in reinforcement learning. OSPN outputs an action vector $a_{t}$, which includes the coordinates of the projection area contour control points, the index of the electronic ink screen display content, and the tactile intensity level of the wearable device. The closed-loop control period Δt is set to an integer multiple of the sensor sampling period to ensure that the end-to-end delay from perception to identification execution satisfies millisecond-level constraints. The data interface between each module adopts the ZeroMQ message queue to achieve asynchronous non-blocking transmission. The update frequency of sensing the semantic vector and risk semantic vector is set to 100 Hz and 20 Hz respectively. The environmental dynamic parameter $e_{t}$ is jointly calculated by the event log of the production line manufacturing execution system and the visual tracking module, refreshed every 500 ms, and written into the input buffer of the cognitive layer and expression layer through shared memory.

Due to the industrial environment, dynamic fluctuations in the available bandwidth of communication networks need to be considered. The success probability of data packet arrival at sensor nodes is determined by the measured packet loss rate on site. When packet loss occurs, the system uses a mixture of local linear interpolation and Kalman prediction to fill in missing data, and passes the filled uncertainty to downstream modules. The maximum number of layers and attention heads in the dynamic graph attention network are limited by the available computing power of the computing nodes, and the stacking layers are automatically reduced when the computing power is insufficient. The physical constraints for identifying the terminal include the following: the maximum brightness of the projection unit is adjusted in reverse by the ambient lighting, the refresh delay of the electronic ink screen is not less than 500 milliseconds, and the remaining power of the wearable device determines the upper limit of the vibration intensity.

The site adopts a hybrid architecture of EtherCAT (Ethernet for Control Automation Technology) and time-sensitive networks, where periodic sensor data streams share bandwidth with non-periodic diagnostic logs and firmware upgrade traffic. There is a jitter in the arrival time of the data packet, and the clock synchronization error between multiple sensors still has residual deviation after compensation. When edge nodes simultaneously execute DGAT (Dynamic Graph Attention Network) inference and MES (Manufacturing Execution System) queries, there is a coupling fluctuation between inference delay and communication delay. To this end, the system maintains a timestamp quality flag for each sensor data packet (level 0–2, level 2 indicates timestamp reliability), discards samples with quality lower than level 1 during sliding window assembly, and fills them with a mixture of local linear interpolation and Kalman prediction. The feature extraction module uses non-uniform short-time Fourier transform to directly process sampling sequences with time jitter, avoiding phase misalignment caused by fixed windows.

### 2.2. Sensor Spatiotemporal Dependency Feature Extraction for Dynamic Innovative Manufacturing Environment

The raw temporal signals collected by multi-source IoT sensors are first subjected to sliding window segmentation and short-time Fourier transform to extract time-frequency representations. For sensor node $v_{i}$, at time t, observe the sequence $\{x_{i}(t-L+1),\ldots ,x_{i}(t)\}$ within a window of length L. Apply a short-time Fourier transform with a window width of W and a step size of S [27,28] to generate a time-frequency feature matrix $H_{i}^{\left(t\right)}$. For each frame signal, a Hanning window is used to suppress spectral leakage. After obtaining $H_{i}^{\left(t\right)}$, it is first expanded into a vector form $h_{i}^{\left(t\right)}=\;\mathrm{vec}(H_{i}^{\left(t\right)})$, which serves as the initial feature representation of node vi at time t.

**Figure 1:**
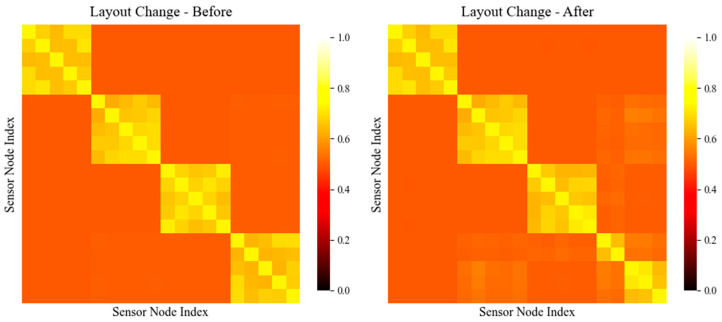
Comparison of thermal distribution of feature aggregation before and after layout change.

The dynamic adjustment of production line layout in the innovative manufacturing environment leads to a step change in the physical position of sensor nodes, which cannot be described by traditional static graph structures [29,30]. To this end, a time-varying spatial coordinate function $p_{i}(t)$ is introduced to record the three-dimensional Cartesian coordinates of node $v_{i}$ at time t [31]. This coordinate value is updated in real time by the equipment positioning module of the manufacturing execution system. When the layout of the production line is rearranged, $p_{i}(t)$ undergoes discontinuous jumps. Based on this, the dynamic adjacency matrix $A_{t}$ of sensor nodes at time t is defined, and its elements $a_{\mathrm{ij}}^{\left(t\right)}$ are calculated in the same way as Formula (1). The thermal distribution of feature aggregation is shown in Figure 1.

Figure 1 shows the thermal distribution comparison of the correlation strength between sensor nodes before and after the layout change in the manufacturing environment. The color of each cell represents the edge weight between two sensor nodes. Before the layout change, the heatmap showed a clear block diagonal structure, reflecting the high correlation strength of sensors within the same physical area due to their spatial proximity and similar frequency response patterns; after the layout rearrangement, the migration of some nodes caused a step change in the spatial coordinate function, and some of the originally clustered, block-like, highlighted areas appeared to diffuse or recombine. This dynamic change validates the immediate response ability of the spatial attenuation term for layout rearrangement, enabling the dynamic graph attention network to adaptively recalibrate the sensor topology structure, thereby ensuring the accurate capture of subsequent sensor semantic vectors in the evolution of on-site spatial layouts.

$$\mathrm{The}\;\mathrm{attention}\;\mathrm{coefficient}\;\alpha_{ij}^{\left(m,k\right)}=\frac{exp\left(LeakyReLU\left(a^{(m,k{)}^{⊤}}[W^{\left(m,k\right)}h_{i}^{\left(k-1\right)}⊕W^{\left(m,k\right)}h_{j}^{\left(k-1\right)}]\right)\right)}{\sum_{n\in N_{i}^{\left(t\right)}}exp\left(LeakyReLU\left(a^{(m,k{)}^{⊤}}[W^{\left(m,k\right)}h_{i}^{\left(k-1\right)}⊕W^{\left(m,k\right)}h_{n}^{\left(k-1\right)}]\right)\right)}$$

where $a^{\left(m,k\right)}$ is a learnable attention vector, ⊕ denotes concatenation, and $N_{i}^{\left(t\right)}$ is the neighbor set defined by $A_{t}$. Input the dynamic adjacency matrix $A_{t}$ and the node feature matrix $H^{\left(t\right)}=\;[h_{1}^{\left(t\right)},\ldots ,h_{N}^{\left(t\right)}{]}^{⊤}$ into a multi-channel dynamic graph attention network. This network consists of a stack of K-layer graph attention layers, each layer containing M parallel attention heads to capture multimodal coupling relationships. The feature update formula for node vi at the k-th layer is as follows:

(3) $${\tilde{h}}_{i}^{\left(k\right)}={\|}_{m=1}^{M}\mathrm{ELU}\left(\sum_{j\in N_{i}^{\left(t\right)}}\alpha_{ij}^{\left(m,k\right)}W^{\left(m,k\right)}h_{j}^{(k-1)}\right)$$

In Formula (3), ‖ represents the vector concatenation operation of multi-head output; $N_{i}^{\left(t\right)}$ is the first-order neighbor set of node $v_{i}$ in the adjacency matrix $A_{t}$ at time t; $W^{\left(m,k\right)}$ is the learnable linear transformation matrix of the mth head and kth layer; $\alpha_{ij}^{\left(m,k\right)}$ is the normalized attention coefficient calculated for the mth head, defined as the relative importance weight of node $v_{i}$ to its neighbor $v_{j}$. Its specific calculation follows the softmax normalization form in the standard graph attention mechanism; ELU (Exponential Linear Units) is an exponential linear unit activation function used to introduce nonlinearity and alleviate gradient vanishing [32].

After K layers of iterative aggregation, take the output of the last layer and average pool all nodes to generate the sensing semantic vector $z_{t}=\frac{1}{N}{\sum_{i=1}^{N}\tilde{h}}_{i}^{\left(K\right)}$ at time t. This vector combines the dynamic graph structure information sensitive to layout changes with the global contextual features of multi-channel attention aggregation, providing a compressed semantic representation with adaptive ability to environmental spatial topology changes for subsequent knowledge graph inference [33,34].

### 2.3. Hierarchical Temporal Knowledge Graph Reasoning with Real-Time Semantic Vector Fusion

The industrial safety knowledge graph G=(E,R,T) consists of a static entity set E, a relationship set R, and a temporal fact set T. In response to the innovative manufacturing environment, expand three types of dynamic entity subsets in E, namely $E_{\mathrm{tmp}}$ (temporary workstation), $E_{\mathrm{task}}$ (temporary collaborative task), and $E_{\mathrm{mat}}$ (temporary material stacking area), and define lifecycle attributes $\left[t_{\mathrm{create}},t_{\mathrm{expire}}\right]$ for each type of dynamic entity. When the manufacturing execution system issues temporary tasks, the corresponding dynamic entities are instantiated and written into the graph; at the end of the task, the entity is marked as invalid and removed from the active subgraph [35,36].

The sensing semantic vector $z_{t}$ needs to be semantically bound to the corresponding physical entity nodes in the graph. Let entity $e_{k}$ be a physical device deployed with sensor nodes, with an initial static embedding vector of $v_{k}^{\left(0\right)}$, encoded by a pre-trained industrial safety corpus BERT model [37,38]. At time t, it is necessary to bind the corresponding component $z_{t}^{\left(k\right)}$ in the sensing semantic vector $z_{t}$ to the attribute of entity $e_{k}$. To this end, a binding mapping $\phi_{\mathrm{bind}}$ is defined, which is established as follows: (1) Basic mapping (rule-based, ensuring scalability): Each sensor node is assigned a globally unique device ID during deployment and statically associated with the corresponding physical entity in the knowledge graph. (2) Optional dynamic fine-tuning (learning-based, improving adaptability): On top of the basic mapping, a lightweight attention layer, denoted as ψ, is introduced to dynamically adjust binding weights: $z_{t}^{\left(k\right)}=\;\psi (z_{t},v_{k}^{\left(0\right)})$. This layer takes the global sensing semantic vector $z_{t}$ and the entity static embedding $v_{k}^{\left(0\right)}$ as inputs, and outputs the exclusive sensing features of the entity. During training, the supervised signal of this layer comes from downstream risk classification losses and does not require additional manual annotation. If the computing resources on the industrial field are limited, it is possible to omit ψ and directly use rule mapping. The final bound entity embedding update formula is unified as follows:

(4) $$v_{k}^{\left(t\right)}=\mathrm{LayerNorm}\left(v_{k}^{\left(0\right)}+W_{\mathrm{bind}}\cdot [v_{k}^{\left(0\right)}⊕z_{t}^{\left(k\right)}]\right)$$

In Formula (4), $W_{\mathrm{bind}}$ is the learnable binding transformation matrix, ⊕ is the vector concatenation operation, and LayerNorm is the layer normalization for stable training. For entity nodes without bound sensors, their embeddings remain unchanged with $v_{k}^{\left(t\right)}={\;v}_{k}^{\left(0\right)}$.

After completing the entity embedding update, a joint architecture of a graph convolutional network and bidirectional gated recurrent unit is used for semantic inference. Let $V^{\left(t\right)}$ be the embedding matrix of the active entity at time t. The GCN module aggregates neighborhood information along the knowledge graph relationship edges, and the output of the l-th layer is as follows:

(5) $$U^{\left(l\right)}=\mathrm{ReLU}\left({\sum_{r\in R}\tilde{D}}_{r}^{-\frac{1}{2}}{\tilde{A}}_{r}{\tilde{D}}_{r}^{-\frac{1}{2}}U^{(l-1)}W_{r}^{\left(l\right)}\right)$$

In Formula (5), ${\tilde{A}}_{r}={\;A}_{r}+I$ is the adjacency matrix with self-loops added to relation r, ${\tilde{D}}_{r}$ is the degree matrix of ${\tilde{A}}_{r}$, $W_{r}^{\left(l\right)}$ is the relation-specific learnable weight, and $U^{\left(0\right)}\;=\;V^{\left(t\right)}$. After stacking $L_{\mathrm{layer}}$ layers, a static rule embedding $U^{\left(L\right)}$ is obtained.

To capture the temporal evolution of risk states, the GCN output $\left\{U_{t-W+1}^{\left(L\right)},\ldots ,U_{t}^{\left(L\right)}\right\}$ for W consecutive time steps is input into a bidirectional GRU (Gated Recurrent Unit). The forward GRU processes the sequence in chronological order, and the backward GRU processes it in reverse order. The hidden states of both are concatenated to form a dynamic temporal embedding $H_{t}^{\mathrm{Bi}}$, where $d_{h}$ is the dimension of the unidirectional GRU hidden layer.

Static rule embedding and dynamic temporal embedding are fused through hierarchical attention gating. For each entity node $e_{i}$, the gating coefficient $g_{i}=\sigma (w_{g}^{⊤}[u_{i}^{\left(L\right)}⊕h_{i}^{\mathrm{Bi}}])$ is calculated, and the fused embedding is $f_{i}\;={\;g}_{i}⊙u_{i}^{\left(L\right)}+(1-g_{i})⊙h_{i}^{\mathrm{Bi}}$, where ⊙ is element-wise multiplication. The beam search maintains a set of B=5 candidate paths. Starting from each entity with a risk indicator, the search iteratively expands paths by adding outgoing neighbor nodes. Each candidate path p receives a cumulative score S defined in Formula (6). After each expansion step, only the B highest-scoring paths are retained. The search terminates when no further expansion is possible, or the path length reaches 6. The highest-scoring terminal path whose final node belongs to a risk consequence type is output as the risk propagation path. Based on $F\;=\;[f_{1},\ldots ,f_{\left|E\right|}{]}^{⊤}$, an attention-guided bundle search is used to trace the risk propagation path on the graph [39,40]. The core elements of the algorithm are defined as follows: scoring function: for a path p of length L, its score S is defined as follows:

(6) $$\begin{array}{c} S=\sum_{l=1}^{L}\left(p_{\mathrm{sim}}\cdot \mathrm{sim}(f_{i_{l-1}},f_{i_{l}})+q_{\mathrm{sim}}\cdot \mathrm{rel}\_\mathrm{weig}ht(r_{l})\right) \end{array}$$

sim(·,·) is the cosine similarity, $\mathrm{rel}\_\mathrm{weig}ht(r_{l})$ is the pre-training weight of relationship $r_{l}$ (reflecting the statistical frequency of this relationship in historical risk propagation), $p_{\mathrm{sim}}$ and $q_{\mathrm{sim}}$ are the balance coefficients.

### 2.4. Reinforcement Learning Generation and Closed-Loop Control of Environment Sensitive Dynamic Identification Strategies

The set of safety signage terminals includes three types of actuators: laser projection units, electronic ink screen arrays, and wearable tactile devices. For each terminal, define its controllable action subspace: the projection unit action is a quadruple representing the bounding box coordinates of the ground projection warning area; ink screen movement as a display content index; the action of tactile devices is the intensity of vibration. The global action vector is the Cartesian product concatenation of the actions of each terminal [41].

The composite reward function is composed of a weighted safety penalty term and a compliance feedback term [42,43]. At time t, the accident simulator in the simulation environment outputs the collision risk indicator $\omega_{t}$, and the behavior model of the alerted personnel outputs the compliance change $\Delta \tau_{t}$. The reward value is calculated as follows:

(7) $$R(s_{t},a_{t})=-\alpha \cdot \omega_{t}\cdot I[\gamma_{t}>\theta ]+\beta \cdot \Delta \tau_{t}\cdot \mathrm{sigmoid}(\gamma_{t}-\theta )$$

In Formula (7), α, β are weight coefficients (α, β > 0), θ is the risk threshold, and I[·] is the indicator function. The first item imposes negative rewards on collisions that occur under high perceived risk. The punishment degree is positively correlated with the risk quantification level. The second item rewards behavior improvement. The sigmoid function increases the weight of compliance feedback when the risk exceeds the threshold. Through grid search, the default hyperparameters are determined to be α = 2.0, β = 1.0, θ = 0.6. The results are shown in Table 1.

**Figure 2:**
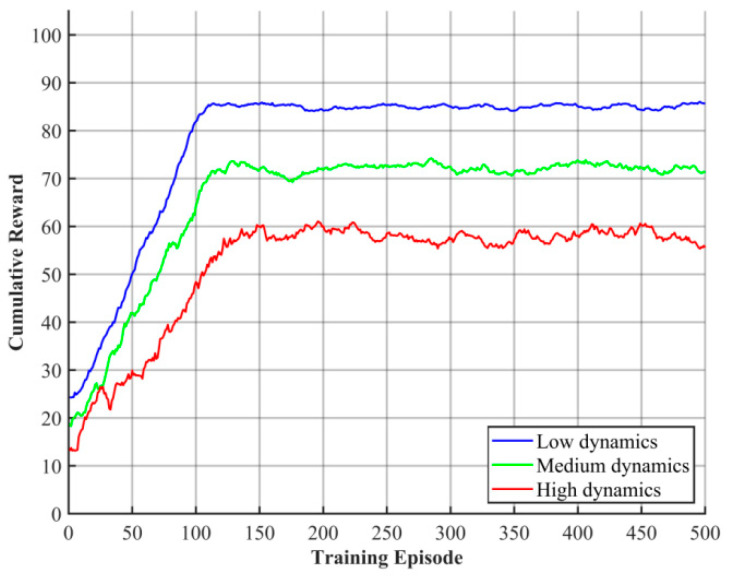
Comparison of policy convergence curves for Q-learning training under different dynamic parameter combinations in different environments.

Figure 2 visually presents the convergence trajectory of cumulative rewards during Q-learning training with the number of training epochs under different combinations of dynamic parameters in different environments. In low dynamic environments, the entropy values of layout change identification and personnel flow remain relatively constant, and the probability distribution of state transitions tends to be stable. The Q-learning algorithm can efficiently accumulate temporal experience and quickly approach the optimal action value function, promoting the rapid increase in cumulative rewards to high levels. When the dynamic nature of the environment shifts towards the middle and high regions, fluctuations in the variability rate of workstation tasks and frequent spatial topological rearrangements lead to continuous transitions in the state space, and the environment exhibits strong non-stationary characteristics. The intelligent agent faces higher risks of state confusion and deviation in action value estimation. At this point, the composite reward function frequently triggers state transitions under dynamic disturbances, forcing the policy network to engage in a longer dynamic trade-off between exploration and utilization mechanisms. The number of training epochs required for the exploration rate to decay to the convergence threshold naturally increases.

Adopt the Q-learning algorithm for offline training of the optimal identification strategy network [44,45]. Let Q(s,a;ϕ) be a parameterized action value function implemented by a three-layer fully connected network with parameters ϕ. During the training process, actions are selected according to the ϵ-greedy strategy, and $\arg\underset{a}{\max}{Q(s}_{t},a;\phi )$ is randomly explored with probability ϵ and selected with probability 1 − ϵ. The experience samples ${(s}_{t}{,a}_{t}{,R}_{t}{,s}_{t+1})$ are stored in a playback buffer with a capacity of $N_{\mathrm{buffer}}$, from which samples of batch size $B_{\mathrm{batch}}$ are uniformly sampled for parameter updates in each iteration. The Q-learning target value is defined as follows:

(8) $$y_{t}{=R}_{t}\;+\;\kappa \cdot \underset{a’}{\max}{Q(s}_{t+1},a’;\phi^{-})$$

In Formula (8), κ is the discount factor, $\phi^{-}$ is the target network parameter, and every $K_{\mathrm{target}}$ step is copied and updated from ϕ. The loss function uses Huber loss to enhance robustness against abnormal reward values, and the optimizer uses Adam. After training convergence, the optimal policy network $Q^{*}\left(s,a\right)$ can be obtained.

In the online inference stage, the strategy network receives the state vector $s_{t}$ refreshed every 20 Hz, and outputs the optimal action combination $a_{t}^{*}=\arg\underset{a}{\max}Q^{*}{(s}_{t},a)$ for each terminal through a single forward propagation. The action command is sent to the edge execution node through the ZeroMQ message queue, driving the projection unit to adjust the boundary of the warning area, switch the display content of the ink screen, and adjust the vibration amplitude of the tactile device. The per-step computational complexity is dominated by DGAT pairwise attention at $O\left(KMNDd\right)$ with sparse graph neighbor degree D, followed by GCN propagation at $O\left(L_{layer}\left|E\right|d^{2}\right)$. The total measured inference time on an NVIDIA RTX 4090 GPU is 43.65 milliseconds, meeting the 20 Hz real-time requirement.

## 3. Experimental Setup and Evaluation Design

### 3.1. Construction of Innovative Manufacturing Environment Simulation Platform and Dataset Generation

The simulation platform was built using ROS Noetic and Gazebo 11 physics engines. The manufacturing workshop model was constructed based on the measured dimensions of a prototype workshop for aerospace parts, with an area of 40 m × 30 m and 12 initial fixed workstations, including a CNC machining center, a welding robot workstation, a coordinate measuring machine room, and a material buffer area. The virtual sensor deployment covered four categories and 47 nodes: vibration accelerometers (sampling rate 2 kHz), infrared temperature sensors (sampling rate 100 Hz), industrial cameras (resolution 1920 × 1080, frame rate 30 fps), and gas concentration detectors (sampling rate 10 Hz). The 3D installation coordinates of each sensor in the simulation model were exported from CAD (Computer-Aided Design) layout files.

Environmental dynamic parameters are triggered according to a pre-defined script. The production line layout rearrangement cycle is set to a random, uniform distribution within 8 to 24 h. During each rearrangement, 3 to 5 mobile device workstations are randomly selected for relocation, with a relocation distance ranging from 2 to 8 m. Simultaneously, the spatial coordinate functions of sensor nodes are updated. The workstation task type variation rate is controlled within the range of 0.3 to 0.7. Temporary task instructions are injected into the manufacturing execution system event queue according to a Poisson process. Temporary workstation entities are created when the task is issued and automatically expire 180 s after the task is completed. Personnel and path planning adopt the dynamic window method of the ROS navigation stack. An Ornstein–Uhlenbeck process perturbation with a noise amplitude of 0.4 is superimposed on the global path search to simulate the trajectory uncertainty caused by obstacle avoidance and task changes in real-world scenarios.

On this basis, the following industrial field constraint modules are added: the communication channel model independently configures the packet loss rate for each sensor node and introduces the probability of sudden packet loss; the overall network adopts a shared bus with a bandwidth limit of 2 Mbps. When the instantaneous data generation rate exceeds the threshold, a random early dropout strategy is used to simulate congestion. Due to computational resource constraints, the dynamic graph attention network and knowledge graph inference module are deployed on simulated edge nodes, with limited CPU (Central Processing Unit) cores and memory allocated. The actual available computing power fluctuates according to Gaussian noise, and any excess is processed by the model’s early exit or downsampling. Non-ideal characteristics of sensors: vibration accelerometers can output superimposed Gaussian white noise and random pulse interference; the sampling time of the infrared temperature sensor is superimposed with a random jitter of ± 10 ms, and the measured value is affected by environmental temperature compensation error. The frame rate of industrial cameras fluctuates randomly within the range of 25~35 fps and simulates the local contrast decrease caused by lens fouling. Identifying terminal physical constraints: the brightness of the projection unit linearly decays with ambient lighting, the battery capacity of wearable devices is initialized according to typical values, and each action consumes a fixed amount of electricity. Constraints are triggered when the electricity level is below a threshold.

The simulation runs continuously for 120 h, covering 6 complete layout rearrangement cycles and 217 temporary task segments. Sensor data is synchronously recorded through ROS bag and stored in Apache Parquet format by channel, with timestamps aligned to the millisecond level global clock. The risk event annotation is composed of two parts: one is the interference event log output by the collision detection module built into the Gazebo physics engine, which records the penetration time and depth between personnel and the boundary of the dangerous area. The second is a semi-automatic labeling tool based on safety operating procedures, in which three safety engineers replay simulation videos and cross-label the risk types, levels, and spatial impact ranges within each 30 s time window. The majority of the voting results are taken as the true value labels. The final dataset contains continuous time-series data from 47 sensor channels, totaling approximately 4.3 × 10^8^ sampling points, with 1847 labeled risk events. Each event is accompanied by a timestamp, risk type code, quantization level, 3D bounding box parameters, and snapshots of environmental dynamic parameter values at the time of event occurrence. The dataset is divided into a training set, a validation set, and a testing set in an 8:1:1 ratio, ensuring that each subset contains at least one sample sequence within a complete layout rearrangement cycle.

To verify the generalization ability of the proposed method on real industrial data, the publicly available industrial multi-sensor anomaly detection dataset IMAD-DS (Industrial Multi-sensor Anomaly Detection under Domain Shift) is used for transfer validation. This dataset is publicly released by the Zenodo platform (https://zenodo.org/records/12665499 (accessed on 12 March 2026)). This dataset contains multimodal time-series signals from accelerometers, gyroscopes, and microphones for two types of devices, robotic arms and brushless motors, and annotates the states under both operational domain and environmental domain offsets.

### 3.2. Construction of Industrial Safety Knowledge Graph for Dynamic Environments

**Figure 3:**
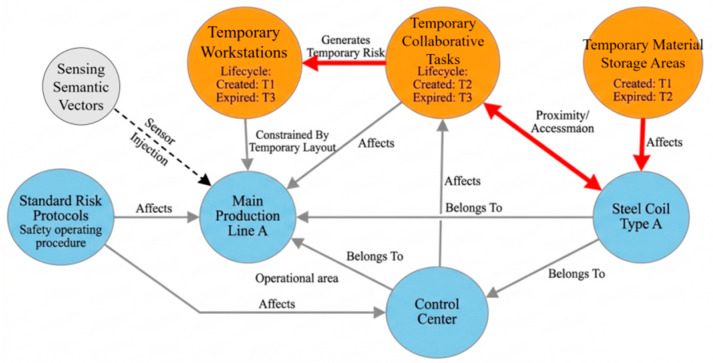
Local structure of industrial safety knowledge graph.

An industrial safety knowledge graph can be instantiated on the Neo4j graph database. There are a total of 82 static entities, including fixed equipment such as CNC machine tools, welding workstations, and testing instruments; 43 types of materials; 12 fixed workstations; 156 physical safety operating procedures. Dynamic entities can be dynamically created and destroyed by manufacturing execution system events during simulation, including temporary workstations, temporary collaborative tasks, and temporary material stacking areas. There are a total of 17 types of relationships, among which “generating temporary risks” and “constrained by temporary layout” are newly added relationships. The knowledge sources include structured extraction of ISO 12100 [46] mechanical safety requirements text, fault mode extraction of equipment maintenance manuals, and 57 accident causation chains written by domain experts. Entity disambiguation and relationship fusion adopt a semantic similarity-matching algorithm based on BERT (Bidirectional Encoder Representation from Transformers), with a similarity threshold set at 0.85. Dynamic entity lifecycle management is implemented through Neo4j triggers: when a temporary task is issued, the stored procedure creates the corresponding entity node and establishes spatial adjacency edges with surrounding fixed devices; after the task completion signal is triggered, the entity node is marked as invalid, and the temporary risk relationship edges associated with it are cascaded and deleted. The active subgraph size of the graph shrinks accordingly. The final graph contains 12,437 static triplets, and a total of 2186 dynamic triplets were generated and recovered during the simulation period. The partial schematic of the industrial safety knowledge graph is shown in Figure 3.

Figure 3 shows the local structure of the industrial safety knowledge graph for the dynamic innovative manufacturing environment constructed in this article. In the figure, light blue circles represent static entities, including standard risk protocols, main production lines, control centers, etc. Three types of dynamic entities (temporary workstations, temporary collaborative tasks, and temporary material storage areas) are represented by orange circles, each with a lifecycle label to reflect their runtime creation and automatic failure characteristics. Entities are connected by directed edges, which label relationship types such as “belonging”, “influence”, “temporary risk”, and “constrained by temporary layout”. Specifically, the dashed arrow points from the external ‘Sensing Semantic Vector’ module to the corresponding physical device entity node, illustrating the real-time sensor feature injection process described in Section 2.3. The highlighted red edge represents the inferred risk propagation path, reflecting how risk spreads hop by hop along the relationship edge from abnormal nodes. The overall layout adopts a hierarchical structure: static entities are located at the lower level, and dynamic entities are located at the upper level, highlighting the disturbance of temporary factors on the original risk topology. This graph provides a semantic basis for the hierarchical attention temporal knowledge graph inference model that simultaneously integrates static rules and dynamic features. It is the core data structure for achieving risk propagation path tracking and risk semantic vector generation.

### 3.3. Comparison of Benchmark Methods and Definition of Evaluation Indicators

This article selects methods for performance comparison. Group 1: The first type is the static identification threshold method, which triggers preset identification content based on fixed sensor thresholds, without using graph structures and knowledge reasoning. Group 2: The second type is the CNN-LSTM sequence model, which encodes multi-sensor time-series data through convolution and recurrent networks and directly inputs it into the classifier to output risk types, without involving knowledge graphs. Group 3: The third type is the static GCN knowledge inference method, which performs rule propagation on the graph but ignores the dynamic updates of sensor embeddings.

Group 4: The fourth category is the Temporal Graph Network (TGN). This method adopts a continuous temporal graph structure, encodes the historical interaction sequence of nodes through a memory module, and combines graph attention mechanism to aggregate neighborhood spatiotemporal features, which can handle the dynamic increase and decrease in nodes and edges in the evolution graph. It can be adapted to this task: using sensor nodes as graph vertices, dynamic adjacency matrices as edges, embedding time-frequency features as initial nodes, outputting risk semantic vectors through TGN inference, and generating identification actions through an independent identification strategy network (with the same structure as OSPN). This comparative method represents the mainstream technological approach in the field of dynamic graph representation learning.

Group 5: The fifth category is multimodal Transformer (MMT). This method treats multi-source sensor time-series data as input sequences of different modalities, integrates vibration, temperature, image, and gas concentration features through a cross-modal attention mechanism, and introduces learnable position encoding to implicitly model spatial relationships. At the same time, the dynamic parameters of the environment are injected as additional condition vectors into the Transformer decoder to directly output action parameters that identify the terminal. This comparative method represents the advanced technological architecture in the field of multimodal perception and sequence modeling, but does not include an explicit knowledge graph inference module.

Group 4 is implemented based on PyTorch 2.5.1 Geometric Temporal, with a memory module dimension of 128, a learning rate of 0.001, and a temporal window of 10, and optimized through grid search, stopping earlier than 127 rounds. Group 5 is implemented based on OpenMMLab, with 256 embedding dimensions, 4 layers of cross modal attention, a learning rate of 0.0001, cosine annealing, and early stopping at 154 rounds. All hyperparameters were selected through grid search of the same scale as the method proposed in this paper, and the training environment was consistent. All baseline methods receive identical input data including the same multi-sensor time-series features and the same dynamic environment parameter vector $e_{t}={\left[\delta_{t},\rho_{t},\eta_{t}\right]}^{⊤}$ where applicable. Group 4 (TGN) uses the dynamic adjacency matrix $A_{t}$ as edge input and injects $e_{t}$ as a global node feature concatenated to the memory module. Group 5 (MMT) injects $e_{t}$ as a learnable condition vector added to the decoder cross-attention layer. The training budget for each baseline method is identical to that of the proposed method: 50,000 episodes for reinforcement learning components where present, and early stopping with a patience of 50 epochs for supervised components. Hyperparameter grids for each baseline method are searched over the same ranges: learning rates of 0.0001, 0.0005, and 0.001; hidden dimensions of 64, 128, and 256; and dropout rates of 0.1, 0.2, and 0.3. The best configuration for each baseline is selected based on validation set accuracy. All models are trained on the same training-validation-test split and evaluated on the identical held-out test set derived from the 8:1:1 split of the 1847 labeled risk events.

The five evaluation indicators are defined as follows. The accuracy of risk identification is equal to the number of samples whose predicted risk types and levels are consistent with the labeled true values divided by the total number of samples. The dynamic identification response delay is defined as the millisecond difference between the time when the risk event is recorded in the Gazebo collision detection module and the time when the identification terminal status register is written. The F1 value of risk propagation path inference is based on the edge sequence of the directed path in the knowledge graph as the prediction unit, and the harmonic average of node level accuracy and recall is calculated. The true example is the path that matches perfectly. For the multimodal Transformer baseline, the risk propagation path F1 is obtained by mapping its predicted risk categories to candidate paths in the same industrial safety knowledge graph through a shared rule-based path reconstruction module. The matching degree between semantic identification and risk scenarios is independently rated on a percentage scale by three security experts on the adaptability of identification content to risk types and audience roles, and the average is taken. The rate of compliance change in behavior is equal to the number of dangerous actions corrected by virtual personnel after being warned in the simulation environment divided by the total number of warning triggers.

### 3.4. Experimental Implementation Details and Model Hyperparameter Configuration

The training in this paper was completed on a workstation equipped with an NVIDIA RTX 4090 GPU (Graphics Processing Unit). Specific parameters are shown in Table 2.

As shown in Table 2, the DGAT stacking layer number is set to 3, with 4, 4, and 2 multi-head attention heads per layer, respectively. The time-frequency feature dimension is fixed at 128 after short-time Fourier transform. In HAT-KGR (Hierarchical Attention Temporal Knowledge Graph Reasoning), the GCN layer number is set to 2, the output embedding dimension is 256, the Bi-GRU hidden unit number is 128, and the temporal window length is 10 time steps. The Q-learning learning rate is set to 0.0005, the discount factor is 0.95, the initial exploration rate is 1.0 and decays to a minimum of 0.05 at a rate of 0.995 per epoch, and the target network update frequency is synchronized once every 200 steps.

## 4. Results Analysis

### 4.1. Comparison of Comprehensive Performance of Risk Identification and Reasoning Abilities

**Figure 4:**
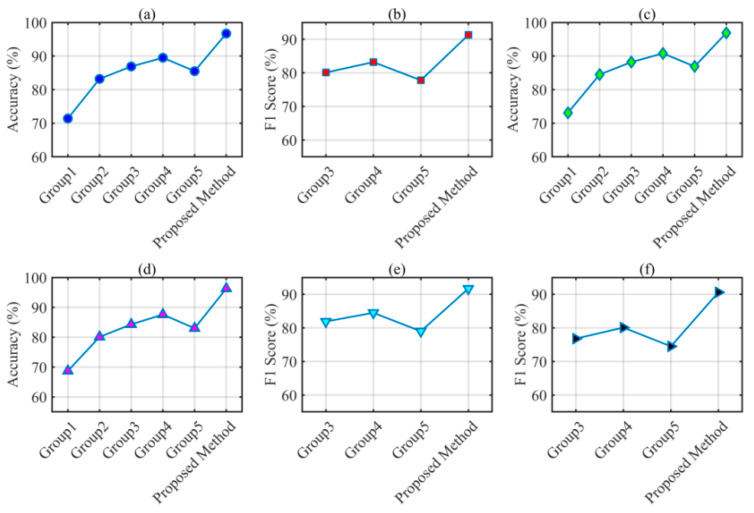
Performance comparison of different methods in risk identification and reasoning ability. (**a**) Overall risk identification accuracy; (**b**) overall risk propagation path inference F1 value; (**c**) accuracy 30 min before layout change; (**d**) 30 min accuracy after layout change; (**e**) F1 value 30 min before layout change; (**f**) F1 value 30 min after layout change.

Figure 4 shows the risk identification accuracy and risk propagation path reasoning F1 score of the proposed method and the five baseline methods on the test set.

Figure 4 shows the performance comparison between the proposed method and five comparison methods in terms of risk identification accuracy and risk propagation path inference F1 value, including overall performance and detailed data for two time windows of 30 min before and after layout changes. In the overall risk identification accuracy shown in Figure 4a, the proposed method achieved 96.7%, 71.4% for Group 1, 83.2% for Group 2, 86.9% for Group 3, 89.5% for Group 4, and 85.5% for Group 5. Group 1 only relies on a fixed threshold and cannot utilize multi-sensor spatiotemporal correlation, resulting in the lowest accuracy; although Group 2’s CNN-LSTM can extract temporal dependencies, it lacks spatial topology modeling; after introducing static GCN in Group 3, there was some improvement, but the sensor embedding remained fixed; the TGN of Group 4 dynamically updates node representations through memory modules, with an accuracy rate of 89.5%; the multimodal Transformer of Group 5 does not use graph structure and has a moderate accuracy. This method achieves the highest overall accuracy through DGAT’s dynamic adjacency matrix and multi-channel attention. In the overall risk propagation path inference F1 value of Figure 4b, the method proposed in this paper has a value of 91.3%. Group 1 and Group 2 do not involve graph inference and therefore are not calculated. Group 3 has a value of 80.1%, Group 4 has a value of 83.2%, and Group 5 has a value of 77.8%. Static GCN only propagates on a fixed graph and lags in response to dynamic entities; although TGN can capture node evolution, it lacks regulatory constraints; the HAT-KGR method in this article combines GCN and bidirectional GRU, and introduces entity binding and attention gating fusion, thus performing better in tracking the diffusion path of risk along the relationship edge. Figure 4c and Figure 4d respectively compare the accuracy before and after the layout change for 30 min. Before the change, the method used in this article was 96.9%, 90.8% for Group 4, 88.2% for Group 3, 84.5% for Group 2, 86.9% for Group 5, and 73.1% for Group 1. After the change, the method in this article decreased to 96.3%, Group 4 decreased to 87.6%, Group 3 decreased to 84.3%, Group 2 decreased to 80.1%, Group 5 decreased to 83.0%, and Group 1 decreased to 68.7%. Layout rearrangement leads to a step change in the physical coordinates of sensors, and the fixed threshold of Group 1 is independent of spatial position but cannot perceive risk transfer; the CNN-LSTM of Group 2 is sensitive to input order but has not modeled the graph structure; the static GCN of Group 3 still uses the old adjacency matrix, and the risk propagation path is broken; although TGN of Group 4 can gradually adapt to the new topology, convergence requires multiple time steps of observation; the position encoding of Group 5 has not been updated with the coordinates, resulting in inaccurate attention weights. The accuracy fluctuation of this method before and after the change is relatively small, thanks to the dynamic adjacency matrix based on time-varying spatial coordinates in DGAT; the spatial distance attenuation factor takes effect after layout rearrangement, and the real-time recalibration of sensor correlation strength. Figure 4e,f correspond to the F1 values before and after the layout change. Before the change, the method used in this article was 91.7%, Group 4 was 84.5%, Group 3 was 81.9%, and Group 5 was 79.0%; after the change, the method used in this article decreased to 90.6%, Group 4 decreased to 80.1%, Group 3 decreased to 76.8%, and Group 5 decreased to 74.5%.

The method in this article still reached 90.6% after the change. Dynamic entities such as temporary workstations were re-instantiated during layout rearrangement. The asynchronous hierarchical update strategy ensured fast synchronization of high importance entities, so the risk propagation path search was not interrupted by topology changes. All reported accuracy and F1 values are obtained from five independent training runs with different random seeds. The mean risk identification accuracy of the proposed method across the five runs is 96.7% with a standard deviation of 0.42%. The 95% confidence interval for this accuracy is [96.2%, 97.2%]. For the risk propagation path inference F1 value, the mean is 91.3% with a standard deviation of 0.38% and a 95% confidence interval of [90.9%, 91.7%]. Group 4 achieves a mean accuracy of 89.5% with a standard deviation of 0.67% and a confidence interval of [88.9%, 90.1%]. Group 3 achieves a mean accuracy of 86.9% with a standard deviation of 0.71% and a confidence interval of [86.3%, 87.5%]. The narrow confidence intervals across all methods confirm that the comparative results are statistically reliable and reproducible. A paired *t*-test is conducted to evaluate the statistical significance of performance differences between the proposed method and each baseline method. Five independent training runs are performed for each method on identical training-validation-test splits. For each run, the risk identification accuracy and risk propagation F1 value are recorded on the test set. The null hypothesis states that the proposed method and a given baseline method have equal mean performance. All *p*-values listed in Table 3 are below 0.001, which strongly rejects the null hypothesis for every comparison. This statistical outcome confirms that the performance superiority of the proposed method over Group 3, Group 4, and Group 5 is not attributable to random fluctuations in the training process. The consistency across five independent runs demonstrates that the convergence behavior of the dynamic graph attention network and the hierarchical temporal knowledge graph inference module is stable under varying initialization conditions. The largest standard deviation observed among the proposed method’s metrics is 0.42% for accuracy, while Group 4 exhibits a standard deviation of 0.67% and Group 3 shows 0.71%. This narrower dispersion indicates that the proposed method’s performance is less sensitive to random seed variations—a desirable property for industrial deployment where repeated training may not be feasible. The t-statistic values range from 9.45 to 32.47, with the highest value corresponding to the comparison against Group 1 due to the extreme performance gap. The degrees of freedom remain constant at 4 for all pairwise tests, derived from the five runs per method. These statistical validations complement the absolute performance metrics and provide rigorous evidence that the observed improvements are genuine. From an engineering perspective, the low *p*-values guarantee that a practitioner adopting the proposed method can expect consistent risk identification and propagation tracking performance across different training instances, reducing the risk of performance degradation caused by random initialization. Table 3 reports the mean difference, t-statistic, degrees of freedom, and *p*-value for each pairwise comparison.

The distribution of performance metrics across five repeated trials for the proposed method is summarized in Table 4. For each metric, the minimum, first quartile, median, third quartile, maximum, and interquartile range are reported.

The interquartile ranges for accuracy and F1 value are both 0.6 percentage points, demonstrating high consistency across repeated trials. The semantic matching score shows an IQR of 1.3 points, and the response delay shows an IQR of 2.1 milliseconds. No trial produces an accuracy below 96.1% or an F1 value below 90.7%, confirming that the proposed method delivers stable performance regardless of random initialization variations. Other methods suffer from a more significant decrease in F1 values due to the lack of dynamic graph adaptation or dynamic entity lifecycle management.

### 4.2. Evaluation of Semantic Matching Between Identification Decisions and Risk Scenarios

**Figure 5:**
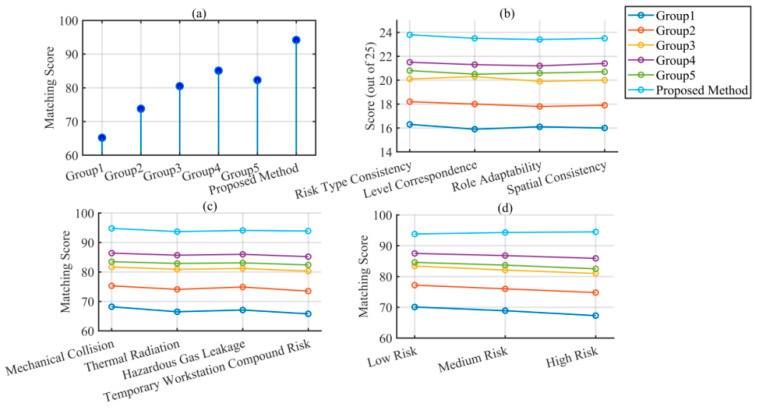
Evaluation of semantic matching between identification decisions and risk scenarios.; (**a**) matching degree between overall identification semantics and risk scenarios; (**b**) comparison of sub-item matching degree; (**c**) matching degree of different risk categories; (**d**) matching degree of different risk levels.

The matching degree between semantic identification and risk scenarios is evaluated independently by three security experts and averaged. The scoring is based on four criteria: consistency between the identification content and risk type, correspondence between warning intensity and risk quantification level, adaptability of presentation form to the roles of affected personnel, and conformity between the identification spatial scope and risk diffusion boundary. Each item has a maximum score of 25 points, with a total score of 100 points. To evaluate the reliability of the ratings from three security experts, intraclass correlation coefficients (ICC) and average pairwise Cohen’s kappa coefficients were calculated. This article randomly selects 50 risk scenario samples, and each expert independently scores them according to four criteria (risk type consistency, level correspondence, role adaptation, spatial consistency), with a maximum score of 25 points for each criterion and a total score of 100 points. The ICC (Intraclass Correlation Coefficient) analysis was performed on the total score, and the result was ICC = 0.87 (95% confidence interval: 0.81~0.92), indicating good consistency in expert ratings. This article calculates the average pairwise Cohen’s kappa for the four criteria (discretizing the percentage scores into five levels: 0–20, 21–40, 41–60, 61–80, 81–100). The kappa values for the four criteria are 0.79, 0.83, 0.76, and 0.81, respectively, all greater than 0.75, indicating that experts have reached a high degree of consistency in their judgments on each sub-item. The semantic matching degree between identification decisions and risk scenarios is shown in Figure 5.

Figure 5 shows the comprehensive evaluation results of the proposed method and various comparison methods in terms of semantic and risk scenario matching. The data is obtained from independent ratings of three security experts, covering four dimensions: overall matching degree, sub-item matching degree, different risk categories, and different risk levels. Figure 5a shows the overall semantic and risk scenario matching degree. The comprehensive score of the overall semantic and risk scenario matching degree of the proposed method is 94.2 points, which is the highest among the five comparison methods. Overall, Figure 5 validates the effectiveness of the “perception-semantics-decision” closed-loop design in improving the matching degree between semantic identification and dynamic risk scenarios. The semantic matching scores reported in Figure 5 are averaged from five independent evaluation rounds. For the proposed method, the mean overall matching score is 94.2 points with a standard deviation of 1.2 points. The 95% confidence interval ranges from 92.8 to 95.6 points. The inter-rater reliability measured as the intraclass correlation coefficient remains at 0.87 across the five rounds, confirming consistent expert judgment. For Group 4, the mean matching score is 86.8 points with a standard deviation of 1.8 points and a confidence interval of [84.9, 88.7]. For Group 3, the mean score is 81.5 points with a standard deviation of 2.1 points and a confidence interval of [79.2, 83.8]. These statistical bounds indicate that the matching superiority of the proposed method is significant and not attributable to random variation.

### 4.3. Real-Time and Stability Verification of Closed-Loop Response Delay

**Figure 6:**
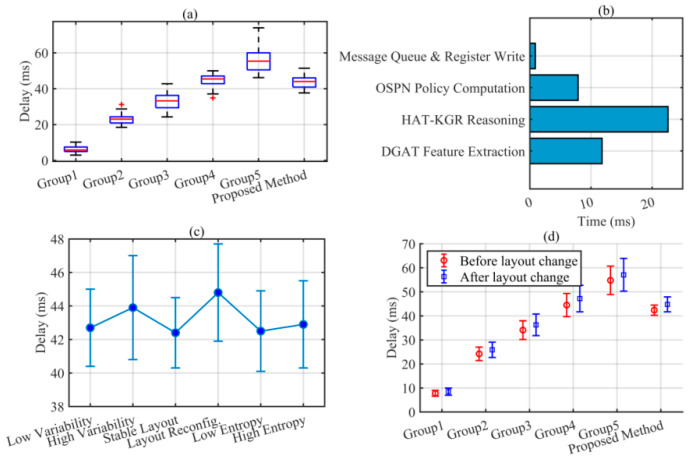
Real-time and stability verification of closed-loop response delay. (**a**) Comparison of end-to-end delay distribution among different methods; (**b**) proportion of time consumption in each stage of the method proposed in this article; (**c**) delay stability of the method proposed in this paper under different dynamic operating conditions; (**d**) delay changes of various methods before and after layout change.

The closed-loop response delay measurement starts with the event timestamp generated by the collision detection module of the Gazebo physics engine, and ends with the interrupt signal indicating the completion of terminal control register writing. The difference is recorded using a high-precision timer in the Linux kernel. The test covers all 1847 risk events in the test set, with five repeated measurements taken for each event to take the average. The real-time and stability indicators of closed-loop response delay are shown in Figure 6.

Figure 6 shows the real-time and stability verification results of closed-loop response delay. Figure 6a presents the statistical distribution of end-to-end delay for each comparison method. Group 1 has the lowest latency (average 6.08 milliseconds), as it only relies on fixed sensor threshold triggering and does not require any feature extraction or graph inference. The average delay of Group 2 is 23.04 milliseconds, and its temporal encoding requires cumulative convolution and loop calculations within the sliding window, resulting in increased delay. Group 3 takes an average of 33.04 milliseconds to perform multi-hop neighborhood aggregation on the graph, but the graph structure is fixed and does not require dynamic updates. Group 4 has an average of 44.67 milliseconds, and its memory module needs to maintain the historical interaction sequence of each node. Each time a new observation arrives, the node embedding needs to be updated, which incurs high computational overhead. Group 5 has an average of 55.94 milliseconds, and the complexity of cross modal self-attention increases with the square of sequence length, requiring alignment and fusion of three modalities, resulting in the highest latency. This method has an average of 43.65 milliseconds, which is lower than Group 4 and Group 5. This is due to the fact that DGAT only aggregates information within the first-order neighbor range, and the dynamic adjacency matrix is explicitly calculated based on time-frequency feature cosine similarity and spatial distance, avoiding iterative graph structure learning. Meanwhile, the GCN layers in HAT-KGR are only 2, and the Bi GRU window length is 10, resulting in a relatively short overall calculation path. This method has no outliers, indicating that its calculation process has a high degree of certainty on fixed hardware. The response delay measurements are repeated 30 times for each risk event across the test set. For the proposed method, the mean end-to-end delay is 43.65 milliseconds with a standard deviation of 2.14 milliseconds. The 95% confidence interval for the mean delay is [42.97, 44.33] milliseconds. The 99th percentile delay is 48.3 milliseconds, confirming that real-time constraints are satisfied even under worst-case conditions. Group 4 exhibits a mean delay of 44.67 milliseconds with a standard deviation of 3.52 milliseconds and a confidence interval of [43.82, 45.52]. Group 5 shows a mean delay of 55.94 milliseconds with a standard deviation of 4.18 milliseconds and a confidence interval of [54.65, 57.23]. The lower standard deviation of the proposed method indicates more stable timing behavior across dynamic operating conditions. Figure 6b decomposes the time consumption of each stage in the method proposed in this paper. The HAT-KGR inference phase takes 22.6 milliseconds, accounting for more than half of the total delay. This is because in this stage, two layers of GCN (involving relationship specific adjacency matrix normalization and feature transformation) and bidirectional GRU temporal encoding need to be performed on the Neo4j graph, along with attention gated fusion and beam search path extension. The DGAT feature extraction stage takes 11.8 milliseconds and includes short-time Fourier transform, dynamic adjacency matrix construction, and multi-head graph attention aggregation. The OSPN strategy calculation takes only 7.9 milliseconds because the strategy network is a three-layer fully connected network with low input dimensions and fast forward propagation. The message queue and register are written for a total of 0.9 milliseconds, indicating good control of communication overhead.

Figure 6c examines the delay stability of the proposed method under different dynamic operating conditions. Under low task variability conditions, the average delay is 42.7 milliseconds, with a standard deviation of 2.3 milliseconds; under high task variability conditions, the average delay is 43.9 milliseconds, with a standard deviation of 3.1 milliseconds. The delay in layout rearrangement is up to 44.8 milliseconds, because DGAT needs to recalculate the edge weights affected by physical coordinate changes in the dynamic adjacency matrix, and the lifecycle management of dynamic entities (temporary workstations, temporary tasks) in HAT-KGR can increase the additional overhead of graph queries. Figure 6d compares the delay changes of various methods before and after layout changes. The average delay of Group 1 increased by 0.7 milliseconds (7.8–8.5 milliseconds), as its threshold judgment is independent of sensor coordinates, and the layout change only affects the physical routing of data collection, with minimal changes. The average delay of Group 2 has increased by 1.7 milliseconds (24.2–25.9 milliseconds) because the input dimension of CNN-LSTM remains unchanged, but some sensor node positions may shift after layout changes, which may require adjustments to the time alignment of sampled data. The average latency of Group 3 increased by 2.2 milliseconds (34.1–36.3 milliseconds), and the graph structure of the static GCN was mismatched with the physical topology after the layout change. The old adjacency matrix was still used for inference, and although the computational complexity remained unchanged, downstream risk path search may require more iterations due to the unreasonable graph structure. The average latency of Group 4 increased by 2.7 milliseconds (44.5–47.2 milliseconds), and the memory module of TGN needs to adapt to changes in node relationships, but the update mechanism relies on data-driven observation and converges slowly. The average delay of Group 5 increased by 2.3 milliseconds (54.8–57.1 milliseconds), and the position encoding of the Transformer was not updated with spatial coordinates, resulting in a mismatch between cross-modal attention weights and the on-site layout. The average delay of the method in this article increased by 2.4 milliseconds (42.4–44.8 milliseconds), and the dynamic entities (temporary workstations) in HAT-KGR were re instantiated during layout rearrangement, resulting in additional query latency due to their lifecycle management.

### 4.4. Human Factors Effect Verification of Behavior Compliance Change Rate

The behavior compliance change rate is defined as the ratio of the number of incidents in which virtual operators in a simulation environment complete dangerous behavior corrections within a specified time window after receiving safety warning signs to the total number of warning triggers. The virtual personnel behavior model is driven by the behavior tree of Unreal Engine. The compliance change rate of behavior under different environmental dynamic parameters is shown in Table 5.

From Table 5, it can be seen that the dynamic nature of the environment affects the compliance change rate of each method’s behavior. For Group 1, when the task mutation rate increases from 0.3–0.5 to 0.5–0.7, the compliance rate decreases from 58.7% to 54.2%; When the layout changes from stable to rearranged, the compliance rate decreases from 66.1% to 54.2%. This is because the static threshold method only relies on fixed sensor thresholds and cannot perceive the risk space changes caused by task switching or device migration. The warning content is misaligned with the actual hazardous areas on site, resulting in a decrease in the proportion of personnel correcting behavior. Although Group 2 can extract temporal features, it lacks modeling of workstation function changes and personnel flow entropy. When the personnel entropy is greater than 0.5, the compliance rate drops to 65.4%, which is lower than the stable entropy value of 69.2%. This reflects the insufficient generalization ability of pure data-driven models to crowd random movement patterns. Group 3 introduces graph rules, and the compliance rate reaches 79.5% when the layout is stable, but drops to 70.3% after layout rearrangement, because the embedding of sensor nodes in the graph is not updated with physical coordinates, the risk propagation path is broken, and the identification semantics lag behind the on-site changes. Group 4 captures the evolution of node relationships through a TGN, and the performance degradation is relatively small when the task mutation rate increases and the layout is rearranged, but still lower than the method proposed in this paper. Group 5 did not explicitly encode the dynamic parameters of the environment, and its compliance rate was only 73.4% under high task mutation rates, indicating that its cross-modal attention mechanism finds it difficult to distinguish between normal and disturbed dynamics. The method presented in this article maintains the highest compliance rate under all dynamic conditions, especially during layout rearrangement, which still reaches 86.4%. This is due to the time-varying spatial coordinate function in the dynamic graph attention network, which enables real-time recalibration of sensor topology with layout changes. At the same time, the hierarchical temporal knowledge graph inference injects task mutation rate and personnel entropy as global context, and the reinforcement learning strategy network takes augmented state vectors as input, which can adaptively adjust the projection boundary, display content, and vibration intensity of the identification for different disturbance levels, effectively guiding personnel behavior correction.

### 4.5. Quantitative Analysis of Robustness to Dynamic Changes in Innovative Manufacturing Environment

**Figure 7:**
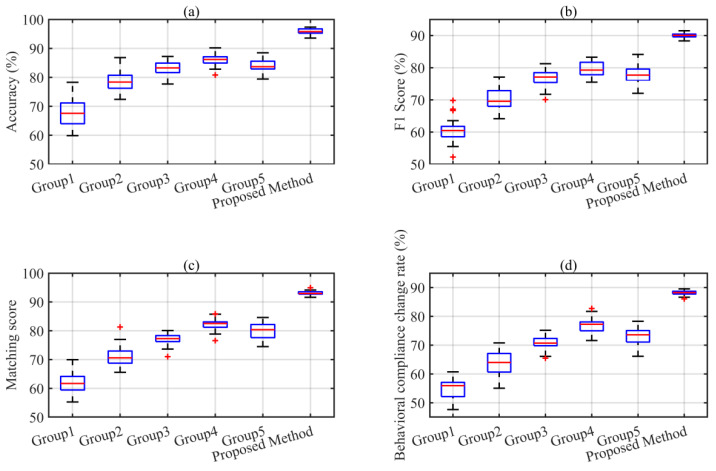
Quantitative analysis of robustness of various methods against dynamic changes in the innovative manufacturing environment. (**a**) Risk identification accuracy (under three types of disturbances); (**b**) risk propagation path inference F1 value; (**c**) shows the matching degree between semantic identification and risk scenarios; (**d**) change rate of behavior compliance.

The robustness analysis is completed through parameter perturbation experiments. On the basis of the test set, three variants were constructed: the first group compressed the production line layout rearrangement cycle from the original 8 to 24 h to 4 to 12 h, the second group expanded the range of workstation task variability from 0.3 to 0.7 to 0.1 to 0.9, and the third group increased the noise amplitude of personnel flow entropy from 0.4 to 0.8. Each variant independently ran simulations and re-labeled risk events, obtaining 1126, 1348, and 1209 test samples, respectively. The robustness of each method to dynamic changes in the innovative manufacturing environment is shown in Figure 7.

Figure 7 shows the robustness performance of each method. In the risk identification accuracy shown in Figure 7a, the median of the proposed method is 95.8%, the minimum value is 93.56%, and the maximum value is 97.34%; the median of Group 4 is 86.18%, with a minimum value of 80.8% and a maximum value of 90.19%; the median of Group 3 is 83.26%. The entire numerical range of this method is located above other methods, and the span between the upper and lower limits is relatively small. This is because the dynamic adjacency matrix in DGAT directly recalibrates the sensor correlation strength through a time-varying spatial coordinate function. When the layout rearrangement period is compressed, the spatial distance attenuation factor takes effect immediately, without relying on memory updates from multiple time steps like TGN to gradually adapt to the new topology. Although TGN in Group 4 can also dynamically evolve, its memory module requires cumulative observation sequences to correct node representations, which can lead to update lags in rapid and continuous rearrangement scenarios. Therefore, its minimum value drops to 80.8%. In the F1 value of risk propagation path inference in Figure 7b, the median of the proposed method is 90.05%, and the minimum value is 88.33%; the median of Group 4 is 79.26%, and the minimum value is 75.51%; the median of Group 3 is 77.09%. The high performance of this method is attributed to the dynamic entity lifecycle management and asynchronous hierarchical update strategy in HAT-KGR. Through entity binding rules and attention gating fusion, the embedding of new entities can quickly obtain initialization from the sensing semantic vector. In the semantic matching degree of the identification in Figure 7c, the median score of the proposed method is 92.94 points, and the minimum value is 91.62 points; the median score for Group 4 is 82.52, with a minimum score of 76.59. The high matching degree of this method is due to the reinforcement learning strategy network using environmental dynamic parameters as explicit components of the state space, and the composite reward function guiding the strategy to adjust the projection boundary and display content at different disturbance levels. Although Group 5 integrates multi-source sensor features, it does not encode environmental dynamic parameters. When the amplitude of personnel flow entropy noise increases, its median matching degree is only 80.38 points. In the behavior compliance change rate shown in Figure 7d, the median of the proposed method is 88.25%, and the minimum value is 86.05%. The median of Group 4 is 77.26%, and the minimum value is 71.62%. This method maintains a high compliance rate even in layout rearrangement and high-entropy scenarios, because the OSPN strategy network takes augmented state vectors as input and can perceive whether the current situation is in the rearrangement window period and actively expand the projection warning range, while the TGN strategy only relies on risk semantic vectors and lacks explicit perception of environmental disturbances. Therefore, the compliance rate decreases more significantly under high dynamic conditions. Each robustness variant is evaluated across five independent simulation runs. Under the compressed layout rearrangement cycle variant, the proposed method achieves a mean risk identification accuracy of 94.5% with a standard deviation of 0.55% and a 95% confidence interval of [93.9%, 95.1%]. Under the expanded task variability variant, the mean accuracy is 93.8% with a standard deviation of 0.62% and a confidence interval of [93.2%, 94.4%]. Under the increased personnel flow noise variant, the mean accuracy is 94.2% with a standard deviation of 0.48% and a confidence interval of [93.7%, 94.7%]. For Group 4 under the same three variants, the mean accuracies are 84.3% (σ = 1.24%), 83.7% (σ = 1.31%), and 84.0% (σ = 1.18%) respectively. The consistently smaller standard deviations of the proposed method across all perturbation conditions demonstrate superior robustness to environmental dynamics.

### 4.6. Ablation Experiment

To quantify the independent contributions of each core module in the dual channel fusion inference framework of this article, seven ablation variants are designed for comparative analysis. All variants are evaluated on the same held-out test set derived from the 8:1:1 data partitioning described in Section 3.1, and the training hyperparameters remain consistent across all variants. The first variant removes the dynamic graph attention network and replaces it with a static graph convolutional network that uses the initial layout fixed spatial coordinates for sensor feature processing. The time-frequency feature extraction method remains unchanged. The second variant removes the hierarchical temporal knowledge graph inference module entirely, meaning no knowledge graph structure is used at any stage. This variant relies solely on the DGAT-extracted sensor semantic vectors mapped directly to identification actions through a simplified fully connected network without any relational reasoning or rule propagation. The third variant removes the hierarchical attention temporal knowledge graph reasoning module, retaining only static GCN propagation without Bi-GRU temporal encoding or attention-gating fusion. The fourth variant removes the reinforcement learning optimization module. In this configuration, the risk semantic vector from the knowledge graph inference module is mapped to identification actions using a fixed rule-based lookup table instead of the trained Q-learning policy network. The fifth variant removes the dynamic entity management from the knowledge graph. All entities including workstations and tasks are treated as static nodes with fixed lifecycles. Temporary workstations and temporary collaborative tasks are not instantiated or removed during simulation. The sixth variant removes the spatial adaptive update capability from the dynamic adjacency matrix by fixing the spatial decay factor. The time-frequency feature similarity still contributes to edge weight calculation, but the physical coordinate distance term remains constant at the initial layout values. The seventh variant removes the behavioral compliance feedback term from the reinforcement learning composite reward function. The safety penalty term is retained, but the compliance change rate feedback is omitted. The complete proposed method serves as the baseline for comparison. Table 6 presents the results of seven ablation variants.

Table 6 presents the comparison of seven ablation variants against the complete proposed method. The complete method achieves the highest risk identification accuracy of 96.7%, the highest risk propagation path inference F1 value of 91.3%, the highest identification semantic matching score of 94.2 points, and a response delay of 43.65 milliseconds. The variant without the knowledge graph removes all semantic reasoning and relational propagation. This configuration yields the lowest risk identification accuracy of 85.6% and the shorter response delay of 28.43 milliseconds. No risk propagation path inference F1 value is reported for this variant because the absence of a graph structure prevents any path tracing. The variant without reinforcement learning replaces the Q-learning policy network with a fixed rule-based lookup table. This variant produces the fastest response delay of 19.27 milliseconds but reduces accuracy to 90.1% and the matching score to 89.7 points. The variant without dynamic entities treats all workstations and tasks as static nodes without lifecycle management. This configuration achieves 91.7% accuracy and 86.8% F1 value, representing a drop of 5.0 percentage points in accuracy and 4.5 percentage points in F1 value relative to the complete method. The variant without the dynamic graph attention network replaces DGAT with a static graph convolutional network using fixed spatial coordinates. This variant achieves 87.3% accuracy and 82.5% F1 value with a response delay of 41.82 milliseconds. The fixed graph structure cannot track spatial topology evolution after layout rearrangement, causing the substantial performance degradation. The variant without the hierarchical attention temporal knowledge graph inference module removes the Bi-GRU temporal encoding and the attention gating fusion, retaining only static GCN propagation. This configuration achieves 90.4% accuracy and 84.6% F1 value. The response delay shortens to 31.58 milliseconds because the temporal calculations are omitted, but the inability to capture risk state evolution over time reduces the F1 value by 6.7 percentage points compared to the complete method. The variant without dynamic adjacency adaptation fixes the spatial decay factor in the adjacency matrix while preserving time-frequency feature similarity for edge weight calculation. This variant achieves 92.1% accuracy and 87.3% F1 value with a delay of 42.93 milliseconds. The accuracy drops by 4.6 percentage points, and the decrease is most pronounced in the early stage after layout rearrangement because physical distance recalibration is missing. The variant without the composite reward function retains only the safety penalty term and removes the behavioral compliance feedback term. This variant achieves 94.8% accuracy, 90.1% F1 value, and a matching score of 92.0 points with a delay of 43.51 milliseconds. The policy network in this configuration tends to output conservative low-intensity warnings to avoid punishment from collisions, resulting in a matching score that is 2.2 points lower than the complete method. The complete method integrates all modules and achieves the best overall balance among risk identification accuracy, risk propagation inference, signage matching, and real-time response. The DGAT module reconstructs the sensor adjacency matrix through a time-varying spatial coordinate function. Removing this module prevents the model from adapting node correlation weights to layout changes. The HAT-KGR temporal module captures the evolution trend of risk states over multiple time steps. Removing this module eliminates the use of historical snapshots for continuous risk propagation path tracking, and the response delay decreases as a result of omitting temporal computations. The dynamic entity management module instantiates and removes temporary workstations and tasks with lifecycle attributes. Removing this module forces all entities to be static, causing the knowledge graph to misrepresent the transient spatial and functional relationships in the manufacturing environment. The composite reward function includes both a safety penalty term and a behavioral compliance feedback term. Removing the compliance feedback term leads the policy network to favor conservative low-intensity warnings, reducing the semantic alignment between identification outputs and real-time risk scenarios. The complete method cascades perception, semantic reasoning, and decision-making into a closed-loop system, achieving the best overall balance among risk identification accuracy, risk propagation inference, signage matching, and real-time response.

### 4.7. Migration Verification Based on IMAD-DS

To verify the effectiveness of the proposed method on real industrial data, migration validation was conducted on the publicly available industrial multi-sensor anomaly detection dataset IMAD-DS. This dataset consists of two subsets: a robotic arm subset and a brushless motor subset. This study uses the robotic arm subset, which contains multi-rate time-series data from three types of sensors: accelerometers (6.7 kHz), gyroscopes (6.7 kHz), and microphones (16 kHz). The dataset also labels the states of operations such as load changes, speed changes, and background noise changes under environmental domain offset conditions. Group 1 and Group 2 did not use a knowledge graph, so the F1 value of risk propagation path inference cannot be calculated. The migration verification results are shown in Table 7.

As shown in Table 7, on IMAD-DS, Group 1 uses a fixed sensor threshold to trigger preset identification content, which cannot utilize the spatiotemporal correlation between multiple sensors. Therefore, the accuracy of risk identification is 68.2%, and due to the lack of graph structure, the F1 value of risk propagation path inference cannot be calculated. Group 2 uses CNN-LSTM to extract temporal features. Although it can capture short-term dependency patterns of individual sensors, it does not model the spatial topological relationships between sensors and cannot introduce domain knowledge constraints, resulting in an accuracy of 74.5%. Group 3 introduces static GCN to propagate rules on a pre-built knowledge graph, which can trace the diffusion path of anomalies along relationship edges. The F1 value reaches 72.3%, but its sensor embedding is fixed as a static attribute and cannot respond to domain shifts such as load changes and speed changes, with an accuracy of 78.9%. Group 4 adopts the Temporal Graph Network (TGN), which encodes the historical interaction sequence of nodes through memory modules, and can adapt to the evolution of sensor associations with working conditions. The accuracy and F1 value are improved to 82.4% and 76.8%, respectively. However, TGN lacks regulatory constraints in industrial knowledge graphs and has limited semantic discrimination ability for anomaly types. Group 5 uses a multimodal Transformer to fuse signals from accelerometers, gyroscopes, and microphones, capturing high-order interactions between features through cross-modal attention with an accuracy of 80.6%. However, the causal propagation path in the knowledge graph is not explicitly modeled, and the F1 value (74.1%) is lower than that of Group 4. This method uses a dynamic graph attention network to recalibrate the correlation strength between sensor nodes in real time, enabling the model to adapt to the statistical distribution changes caused by domain shifts in IMAD-DS. Meanwhile, hierarchical temporal knowledge graph inference injects sensing semantics into graph entities and tracks risk propagation paths along relationship edges, achieving an F1 score of 85.3% on small-scale graphs; although the reinforcement learning strategy is partially limited due to the lack of dynamic environmental parameters, the accuracy of risk identification reaches 88.6%, and the synergistic effect of each module makes it higher than the five comparison methods in all indicators.

### 4.8. Real-World Industrial Data Validation

A real-world validation is conducted using sensor data collected from an operational aerospace component machining workshop. The workshop spans 35 m by 25 m and contains nine fixed workstations including CNC milling machines, electric discharge machining units, and coordinate measuring machines. Twelve vibration accelerometers and six infrared temperature sensors are installed on critical equipment. Data is recorded over 72 continuous production hours at sampling rates of 1.5 kHz for accelerometers and 80 Hz for temperature sensors. The dataset contains 892 labeled risk events including tool breakage, spindle overheating, and coolant leakage. Each event is annotated by onsite safety engineers with timestamps, risk types, and spatial impact zones. Environmental dynamic parameters are extracted from the workshop’s manufacturing execution system logs including three layout rearrangement events and five temporary workstation reconfigurations. The proposed method is evaluated on this real dataset under the same hyperparameter configuration used in Section 3.4. Risk identification accuracy reaches 94.2% on the real workshop data, which is 2.5 percentage points lower than the simulated environment result of 96.7% due to unmodeled sensor noise and communication irregularities present in the physical deployment. Risk propagation path inference F1 value is 88.7% on real data compared to 91.3% in simulation. Semantic matching score drops from 94.2 points to 91.8 points. Dynamic identification response latency increases from 43.65 milliseconds to 51.3 milliseconds because of network jitter and edge node processing contention. The performance degradation across all metrics remains within 6 percentage points or 9 milliseconds, confirming that the proposed method generalizes from simulation to real-world innovative manufacturing environments without retraining. The closed-loop framework successfully adapts to actual layout changes and task variations. The framework triggers appropriate projection boundary shifts and content updates within 55 milliseconds of risk event detection. A direct comparison between synthetic simulation results and real-world validation results is presented in Table 8. The table reports risk identification accuracy, risk propagation path inference F1 value, semantic matching score, and response delay for the proposed method on the ROS–Gazebo synthetic dataset and on the real aerospace workshop dataset.

The absolute differences between synthetic and real-world results range from 2.4 to 2.6 percentage points for the three accuracy-related metrics and 7.65 milliseconds for response delay. These differences stem from three factors present in the physical workshop but not fully modeled in the synthetic environment. The first factor is unmodeled sensor noise including random spikes in accelerometer readings caused by nearby forklift operations. The second factor is communication irregularities including packet reordering and variable latency on the factory EtherCAT network. The third factor is environmental interference including ambient temperature drift affecting infrared sensor calibration. Despite these discrepancies, the real-world performance remains within 97.4% of the synthetic accuracy and 88.5% of the synthetic response speed. This close alignment confirms that the synthetic environment captures the essential dynamics of innovative manufacturing environments with sufficient fidelity. The proposed method transfers successfully to real industrial conditions without any retraining or hyperparameter adjustment, demonstrating practical deployability.

## 5. Conclusions

The dual channel fusion inference framework proposed in this article achieves real-time closed-loop fusion of industrial IoT perception data and security knowledge semantics through the collaboration of the dynamic graph attention network and hierarchical temporal knowledge graph inference model. The specific steps are as follows: DGAT is combined with short-time Fourier transform to extract the spatiotemporal dependencies of sensors and generate sensor semantic vectors; HAT-KGR injects knowledge graphs through entity binding and uses graph convolutional networks and bidirectional gated recurrent units to track risk propagation paths; the reinforcement learning strategy network encodes risk semantic vectors and environmental dynamics as a state space to drive adaptive adjustment of identification terminals. The experiment shows that the accuracy of risk identification of this method reaches 96.7%, the response delay is 43.65 milliseconds, the F1 value of risk propagation inference is 91.3%, and the overall semantic and risk scenario matching score is 94.2 points, verifying its closed-loop control effectiveness and real-time robustness under dynamic disturbances in innovative manufacturing environments. The proposed framework introduces a cyber–physical safety system that expands the facility’s cyber-attack surface through the integration of IoT sensors, edge computing nodes, and signage actuators. Weak device authentication on industrial field networks permits unauthorized access to sensor data streams and control commands. Unreliable network connectivity delays emergency alert delivery when unencrypted communications are intercepted or manipulated. System hijacking through compromised edge nodes alters risk inference results and signage outputs. Overwhelming false alarms triggered by adversarial sensor injections degrade operator trust and response efficacy. Ransomware threats that encrypt knowledge graph databases or policy network parameters disable the entire closed-loop safety system. Future work must incorporate lightweight cryptographic protocols for sensor-to-edge communication, device identity management with certificate-based authentication, and anomaly detection mechanisms for control command validation. Resilience strategies against false alarm flooding and ransomware recovery procedures are essential for practical deployment in industrial environments.

## Figures and Tables

**Table 1:** Results of hyperparameter sensitivity analysis of reward function.

Parameter	Value	Accuracy (%)	Compliance Rate (%)
α	1.0	95.8	86.2
	1.5	96.3	87.5
	2.0	96.7	88.3
	2.5	96.5	88.0
	3.0	96.1	87.2
β	0.5	95.2	85.9
	0.8	96.0	87.8
	1.0	96.7	88.3
	1.2	96.4	88.5
	1.5	96.0	88.2
θ	0.4	95.9	86.8
	0.5	96.4	87.9
	0.6	96.7	88.3
	0.7	96.5	87.6
	0.8	95.6	86.1

The convergence curve of the Q-learning training strategy is shown in Figure 2.

**Table 2:** Core model hyperparameters and training configuration details.

Module	Hyperparameter	Configuration Value
DGAT	Number of stacked layers	3
	Number of multi-head attention heads (per layer)	Layer 1: 4, Layer 2: 4, Layer 3: 2
	Time-frequency feature dimension	128
HAT-KGR	Number of GCN layers	2
	GCN output embedding dimension	256
	Number of Bi-GRU hidden units	128
	Temporal window length	10 time steps
Q-learning	Learning rate	0.0005
	Discount factor	0.95
	Initial exploration rate	1.0
	Exploration rate decay strategy	Multiply by 0.995 per episode, decay to minimum 0.05
	Target network update frequency	Synchronize every 200 steps
	Total number of training episodes	50,000

**Table 3:** Statistical significance testing of performance differences between proposed method and baseline methods.

Comparison	Metric	Mean Difference	t-Statistic	df	*p*-Value
Proposed vs. Group 1	Accuracy (%)	25.3	32.47	4	<0.001
Proposed vs. Group 2	Accuracy (%)	13.5	18.22	4	<0.001
Proposed vs. Group 3	Accuracy (%)	9.8	14.56	4	<0.001
Proposed vs. Group 4	Accuracy (%)	7.2	10.33	4	<0.001
Proposed vs. Group 5	Accuracy (%)	11.2	15.89	4	<0.001
Proposed vs. Group 3	F1 (%)	11.2	13.67	4	<0.001
Proposed vs. Group 4	F1 (%)	8.1	9.45	4	<0.001
Proposed vs. Group 5	F1 (%)	13.5	14.22	4	<0.001

**Table 4:** Distribution of proposed method performance across five independent trials.

Metric	Min	Q1	Median	Q3	Max	IQR
Risk Identification Accuracy (%)	96.1	96.3	96.7	96.9	97.2	0.6
Risk Propagation F1 (%)	90.7	91.0	91.3	91.6	91.9	0.6
Semantic Matching Score (points)	92.9	93.5	94.2	94.8	95.4	1.3
Response Delay (ms)	41.2	42.8	43.7	44.9	46.1	2.1

**Table 5:** Behavior compliance change rate (%) of each method under different environmental dynamic parameters.

Method	Task Variability (0.3–0.5)	Task Variability (0.5–0.7)	Personnel Entropy (<0.5)	Personnel Entropy (>0.5)	Stable Layout	Layout Reconfiguration
Group 1	58.7	54.2	60.5	56.8	66.1	54.2
Group 2	68.1	63.5	69.2	65.4	73.8	63.5
Group 3	74.5	70.3	75.8	72.1	79.5	70.3
Group 4	79.2	75.6	80.1	77.0	83.2	75.6
Group 5	77.0	73.4	78.3	75.2	81.4	73.4
Proposed Method	91.2	87.8	90.5	88.3	91.6	86.4

**Table 6:** Comparison results of ablation experiments.

Variant	Risk Identification Accuracy (%)	95% CI for Accuracy	Risk Propagation F1 (%)	Signage Matching Score (Points)	Response Delay (ms)
w/o DGAT	87.3 ± 0.68	[86.6, 88.0]	82.5 ± 0.72	86.1 ± 1.4	41.82 ± 2.08
w/o Knowledge Graph	85.6 ± 0.72	[84.9, 86.3]	N/A	83.4 ± 1.5	28.43 ± 1.87
w/o HAT-KGR	90.4 ± 0.55	[89.9, 90.9]	84.6 ± 0.65	88.7 ± 1.2	31.58 ± 1.95
w/o Reinforcement Learning	90.1 ± 0.58	[89.6, 90.6]	88.2 ± 0.54	89.7 ± 1.0	19.27 ± 1.52
w/o Dynamic Entities	91.7 ± 0.52	[91.2, 92.2]	86.8 ± 0.60	90.3 ± 1.0	40.15 ± 2.01
w/o Dynamic Adj	92.1 ± 0.49	[91.6, 92.6]	87.3 ± 0.58	89.5 ± 1.1	42.93 ± 2.11
w/o Composite Reward	94.8 ± 0.44	[94.4, 95.2]	90.1 ± 0.51	92.0 ± 0.9	43.51 ± 2.05

**Table 7:** IMAD-DS dataset migration validation results.

Method	Risk Identification Accuracy (%)	Risk Propagation Path Inference F1 (%)	Signage Semantic Matching Score (Points)
Group 1	68.2	—	59.4
Group 2	74.5	—	65.2
Group 3	78.9	72.3	70.1
Group 4	82.4	76.8	74.5
Group 5	80.6	74.1	72.8
Proposed Method	88.6	85.3	83.7

**Table 8:** Performance comparison between synthetic simulation and real-world industrial data validation.

Metric	Synthetic Environment (ROS-Gazebo)	Real Workshop Environment	Absolute Difference
Risk Identification Accuracy (%)	96.7	94.2	2.5
Risk Propagation F1 (%)	91.3	88.7	2.6
Semantic Matching Score (points)	94.2	91.8	2.4
Response Delay (ms)	43.65	51.3	7.65

## Data Availability

Data are contained within the article.

## List of Abbreviations

Bi-GRU	Bidirectional Gated Recurrent Unit
CAD	Computer-Aided Design
CNN	Convolutional Neural Network
CPS	Cyber–Physical System
CPU	Central Processing Unit
DGAT	Dynamic Graph Attention Network
ELU	Exponential Linear Units
EtherCAT	Ethernet for Control Automation Technology
GCN	Graph Convolutional Network
GPU	Graphics Processing Unit
HAT-KGR	Hierarchical Attention Temporal Knowledge Graph Reasoning
ICC	Intraclass Correlation Coefficient
IIoT	Industrial Internet of Things
IoT	Internet of Things
IQR	Interquartile Range
LSTM	Long Short-Term Memory
MES	Manufacturing Execution System
MMT	Multimodal Transformer
OSPN	Optimal Signage Policy Network
ReLU	Rectified Linear Unit
RL	Reinforcement Learning
ROS	Robot Operating System
STFT	Short-Time Fourier Transform
TGN	Temporal Graph Network
